# A review of the biology of calcium phosphate sequestration with special reference to milk

**DOI:** 10.1007/s13594-014-0177-2

**Published:** 2014-07-16

**Authors:** Samuel Lenton, Tommy Nylander, Susana C. M. Teixeira, Carl Holt

**Affiliations:** 1 EPSAM, Keele University, Keele, Staffordshire ST5 5BG UK; 2 Institut Laue Langevin, 71 avenue des Martyrs, CS 20156, 38042 Grenoble cedex 9, France; 3 Department of Physical Chemistry, Lund University, P.O. Box 124, S-221 00 Lund, Sweden; 4 Institute of Molecular, Cell and Systems Biology, University of Glasgow, RB413A Level B4 Joseph Black Building, Glasgow, G12 8QQ UK

**Keywords:** Amorphous calcium phosphate, Blood, Saliva, Milk, Urine, Bone, Tooth

## Abstract

In milk, a stable fluid is formed in which sequestered nanoclusters of calcium phosphate are substructures in casein micelles. As a result, calcium and phosphate concentrations in milk can be far in excess of their solubility. Variations of calcium, phosphate and casein concentrations in milks, both within and among species, are mainly due to the formation of the nanocluster complexes. Caseins evolved from tooth and bone proteins well before the evolution of lactation. It has therefore been suggested that the role of caseins in milk is an adaptation of an antecedent function in the control of some aspect of biomineralisation. There is new evidence that nanocluster-type complexes are also present in blood serum and, by implication, in many other closely related biofluids. Because such fluids are stable but nevertheless supersaturated with respect to the bone and tooth mineral hydroxyapatite, they allow soft and mineralised tissues to co-exist in the same organism with relative ease. An appreciable concentration of nanocluster complexes exists in fresh saliva. Such saliva may stabilise tooth mineral and help to repair demineralised lesions. In the extracellular matrix of bone, nanocluster complexes may be involved in directing the amorphous calcium phosphate to intrafibrillar spaces in collagen where they can mature into oriented apatite crystals. Thus, evidence is accumulating that calcium phosphate sequestration by phosphopeptides to form equilibrium complexes, first observed in milk, is more generally important in the control of physiological calcification.

## Introduction

Research into the mechanisms of biomineralisation and the chemistry of the mineral phases has been the subject of a number of excellent recent reviews (George and Veis 2008; Wang and Nancollas 2009; Dorozhkin 2011). Hard tissues include bone, cementum, dentine and enamel and contain a basic calcium phosphate mineral called apatite, or hydroxyapatite, named by geologists from the Greek word *apate* meaning deceive because of the variable crystal habit and composition of apatite rocks. Soft tissues and biofluids, by contrast, contain little or no mineral in their normal, physiological state. Soft tissues can become mineralised and hard tissues can become demineralised as a result of degenerative, dysfunctional or diseased conditions, but normally, tissues remain stably mineralised or unmineralised even though they are permeated by the same extracellular fluid. The problem of the easy co-existence of soft and mineralised tissues in the same organism can be stated easily in terms understandable to a chemist. For the hard tissue to stay mineralised, the permeating biofluid must be supersaturated with respect to the hard tissue mineral, and yet, for the soft tissues and biofluids to remain unmineralised, the permeating biofluid must be stable.

A solution to the problem of the stability of biofluids must have been found more than about 500 Ma ago when the first calcium phosphate mineralised tissues appear in the fossil record. These include the cone-shaped denticles or teeth of jawless fishes called conodonts. By about 50 Ma later, homologues of the modern hard tissue types were established. For example, the bone-like head plates of placoderms may have allowed sense organs, mounted on this stable platform, to build up a three-dimensional picture of their environment. Concomitantly, a paralogous group of secreted, calcium (phosphate)-binding phosphoproteins evolved, called SCPPs (Kawasaki et al. 2004; Kawasaki and Weiss 2003). Members of this group of proteins are involved in every aspect of biomineralisation. One of the earliest members of the group of SCPPs is osteopontin which has a very wide occurrence in species, tissues and biofluids (Mazzali et al. 2002).

Caseins are also members of the SCPP group, and it has been known for many years that the colloidal calcium phosphate of casein micelles allows stable milks to be formed, containing much higher calcium and phosphate concentrations than are permitted by the solubility of inorganic calcium phosphates at milk pH. Because of the ready availability of milk and the high concentrations of the caseins responsible for its stability, we have been able to gain a deeper understanding of the reasons for its stability than is available for any other biofluid. These insights have led to an improved understanding of the sources of stability in some other biofluids.

In this review, we summarise some of the evidence of calcium phosphate sequestration dairy chemistry in biofluids and soft and hard tissues. We believe that a dialogue between the dairy chemistry and biomineralisation communities would be to their mutual benefit.

## Basic science of amorphous calcium phosphate sequestration by phosphoproteins

### Ostwald rule of stages

At physiological pH, calcium phosphate does not precipitate directly from solution as the most thermodynamically stable phase, hydroxyapatite. Instead, it passes through a number of unstable or metastable stages from the most soluble to the least. Typically, an initial and highly unstable amorphous calcium phosphate (ACP) phase, ACP-1, is succeeded by a more stable and less soluble amorphous phase, ACP-2 (Christoffersen et al. 1990). It has been shown that both amorphous phases had the same ratio of calcium to phosphate of 1.35 which is significantly lower than that of a basic tricalcium phosphate (Ca/P = 1.5) but above that of an acidic dicalcium phosphate (Ca/P = 1.0). The chemical formula Ca(PO_4_)_0.52_(HPO_4_)_0.22_ is consistent with Ca/P = 1.35 (Christoffersen et al. 1990). In similar studies (Termine and Eanes 1972; Meyer and Eanes 1978; Wuthier et al. 1985; Holt et al. 1989), the Ca/P ratio of ACP has been reported to be as high as 1.5 and as low as 1.18, demonstrating that a range of more-or-less acidic ACP phases are possible. Subsequently, poorly crystalline, impure and non-stoichiometric phases are formed, the best characterised of which is octacalcium phosphate. The mineralised tissues of bone, cementum and dentine all contain poorly crystalline calcium phosphates of these sorts, but enamel comprises highly crystalline prisms of almost pure hydroxyapatite.

### Effect of phosphopeptides

Phosphoproteins and phosphopeptides can affect every aspect of the precipitation process. For example the calcium-sensitive bovine caseins, even at very low concentrations, can extend the lag time before precipitation begins and slow down the rate of maturation of the amorphous state. Phosphopeptides that contain three or more phosphorylated residues (usually seryl) in a short sequence are said to have a phosphate centre (PC). If short, acidic, PC-containing peptides are present in stoichiometric excess over the amount of amorphous calcium phosphate that forms from a supersaturated solution, the solution remains apparently unchanged but contains calcium phosphate nanoclusters with an outer shell of the phosphopeptides. Further investigations have shown that the nanoclusters are equilibrium particles with a defined size and composition. In all other circumstances, a precipitate will develop, although the solution may remain in a metastable state for some time. Certain highly phosphorylated phosphoproteins such as the C-terminal regions of osteopontin or phosphophoryn appear to be able to nucleate hydroxyapatite (Boskey et al. 2010).

### Ion activity product

The ion activity product has played an important part in the science of calcium phosphate sequestration. For a generic chemical formula of an amorphous calcium phosphate, containing both HPO_4_^2 −^ and PO_4_^3 −^ (Combes and Rey 2010)

1 $$ {\mathrm{Ca}}^{2+}+ y{\mathrm{HPO}}_4^{2-}+\frac{2\left(1- y\right)}{3}{\mathrm{PO}}_4^{3-}\leftrightarrow {\left[\mathrm{Ca}{\left({\mathrm{HPO}}_4\right)}_y{\left({\mathrm{PO}}_4\right)}_{\frac{2\left(1- y\right)}{3}}\right]}_{\mathrm{solid}} $$

the corresponding ion activity product is

2 $$ {K}_{\mathrm{S}}=\left\{{\mathrm{Ca}}^{2+}\right\}{\left\{{\mathrm{HPO}}_4^{2-}\right\}}^y{\left\{{\mathrm{PO}}_4^{3-}\right\}}^{2\left(1- y\right)/3} $$

where the quantities in curly brackets are activities and the value of *y* determines how acidic the amorphous phase is. For example, a basic tricalcium phosphate has *y* = 0, the ACP-1 and ACP-2 phases of Christoffersen et al. have *y* = 0.22 and the acidic dicalcium phosphates have *y* = 1.0. We have used the ion activity product to show that sequestered amorphous calcium phosphates can be acidic or basic salts depending on the sequestering phosphopeptide. For a solid phase in equilibrium with the continuous phase, the corresponding ion activity product is invariant with respect to variation in the composition and pH of the fluid. Conversely, if an invariant ion activity is found in a fluid, then the nature of the solid phase (for example, nanoclusters, amorphous or crystalline calcium phosphates) with which it is in equilibrium can be inferred. This is true even if the phase is present at a vanishingly small concentration. J.C. Elliot has written an advanced treatise on calcium phosphates which includes values for the solubility products of all the biologically relevant phases (Elliot 1994).

### Application of a thermodynamic model to physiological fluids

The calculation of the ion equilibria in biological fluids on the assumption that all components are in solution as simple ions and ion complexes is well established (for references, see Holt et al. 2014). A tractable theory of the additional calculation of the partition of salts between sequestered and free forms in a physiological fluid containing phosphopeptides can be formulated on the assumption that there is a further equilibrium reaction in which phosphopeptides bind strongly to amorphous calcium phosphate to form a stoichiometric complex. Experimental evidence confirms that the sequestered complexes have a constant composition and equilibrium size and the solution exhibits an invariant ion activity product (Little and Holt 2004; Holt et al. 2009). A further assumption is that all the phosphate centres have the same capacity to sequester amorphous calcium phosphate and it may sometimes be necessary to know the ion binding properties of free phosphate centres.

The simple ion equilibria calculations require the equilibrium constants for all the ion complexes likely to be present (Holt et al. 1981; Mekmene et al. 2009, 2010; Lyster 1981; Rice et al. 2010; Holt 2004). The additional parameters needed for the partition calculation are listed in Table 1. Each can be determined in independent experiments on well-defined solutions in which there is a known PC-containing phosphopeptide. Because of this and the fact that no fitting process with adjustable parameters is used to describe the unknown fluid, we can describe the calculation as ab initio.

**Table 1:** Parameters needed for the ab initio calculation of the partition of salts in a fluid

Parameter	Description
*C*_1_, *C*_2_, *C*_*n*_	Concentrations of the *n* principal salts
*R*_*C*1_, *R*_*C*1,_ *R*_*Cn*_	Molar ratio of each salt constituent to PC in the sequestered complex
[PC]	Total concentration of phosphate centres
*y*	Acidity parameter defined in Eq. (1)
*K*_S_	Solubility constant or invariant ion activity product
*v*(*C*_1_, *C*_2_, *C*_*n*_)	Isotherm describing the simultaneous binding of the *n* principal salts to a free PC

Application of the model to simple nanocluster solutions and to milk has been described (Little and Holt 2004; Holt 2004; Holt et al. 2009).

### Stability diagram of biofluids

**Fig. 1:**
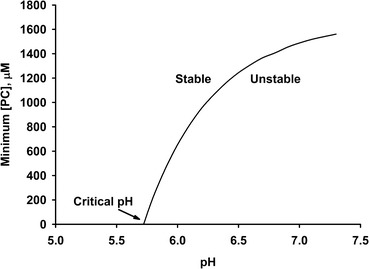
Stability diagram for a bovine milk-like fluid

A special case of the calculation is for the condition that the concentration of phosphate centres is the minimum needed to sequester the quantity of calcium phosphate formed at equilibrium. The variation of the minimum concentration with pH has been used to calculate the stability diagram for a variety of biofluids (Holt et al. 2014). An example of the stability diagram for a bovine milk-like fluid is shown in Fig. 1.

The stability diagram defines areas of stability and instability. Below a certain pH, called the critical pH, no phosphopeptides are needed to make the solution stable. As pH increases, the minimum concentration of phosphate centres increases in a curvilinear manner up to a plateau value somewhere above pH 8. Close to the boundary line, on the higher pH side, is an ill-defined area of metastability where precipitation may be delayed for some time.

If the solution is a biofluid and if the critical pH is below the physiological pH, then it will be liable to precipitate calcium phosphate. Nevertheless, if it contains a sufficient concentration of a sequestering phosphopeptide, it can be perfectly stable, even at the physiological pH. At equilibrium it will contain nanoclusters of sequestered calcium phosphate. For the example calculation shown in Fig. 1, the physiological pH is about 6.7 where a considerable concentration of phosphate centres is needed to ensure stability.

## Application to milk

In milk, the main source of phosphate centres is the calcium-sensitive caseins, but all caseins contain adhesive sequences to promote mutual association. As a result, all milks contain colloidal particles called casein micelles instead of individual nanoclusters. Nevertheless, the casein micelle can be regarded as an assembly of nanoclusters. For example, a bovine milk casein micelle with a radius of 100 nm is an assembly of about 800 nanoclusters and each nanocluster is formed from peptides containing about 50 phosphate centres (Holt et al. 2003). The average spacing between the nanoclusters is about 18 nm which has been mistaken, in the past, for the diameter of protein submicelles (McMahon and Oommen 2012).

**Fig. 2:**
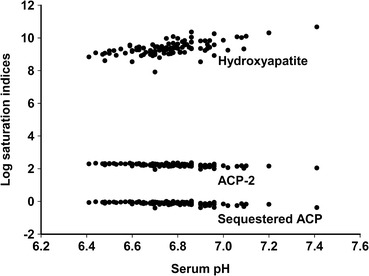
Saturation indices of bovine milk sera for hydroxyapatite (Holt 1982), ACP-2 (Christoffersen et al. 1990) and ACP sequestered by casein phosphopeptides (Little and Holt 2004). Calculations used the data of individual milk samples in early, middle and late lactation (White and Davies 1958)

Figure 2 shows that cow’s milk serum is highly supersaturated with respect to hydroxyapatite and there is no invariant ion activity product for this phase. There is a nearly invariant ion activity product for ACP-2, the more stable phase of amorphous calcium phosphate, but the serum is supersaturated with respect to ACP-2. Milk serum shows virtually the same invariant ion activity product as a solution of casein nanoclusters (Little and Holt 2004).

When the quantitative predictions of the ab initio calculations of the partition of milk salts are compared to experiment for individual cow milk samples with pH values in the physiological range, the agreement between theory and experiment is very satisfactory (Holt 2004). The extension of the theory to consider some of the non-physiological conditions that are encountered during routine milk processing operations has also been attempted. These include the effects of the adjustment of pH, addition or removal of salts, addition of caseins, concentration and dilution on the partition of salts and the dissociation of individual caseins from the casein micelle. Presently beyond the capabilities of the model are the partition of micronutrients and radionuclides, the effect of temperature and pressure and the consequences of all these manipulations on the aggregation or gelation of milk proteins.

## Application to other physiological fluids

Nearly all physiological fluids are supersaturated with respect to hydroxyapatite and some other crystalline calcium phosphate phases. As a result, artificial biofluids that closely resemble their natural counterparts in salt composition and pH are unstable and will readily precipitate calcium phosphate. They, and natural biofluids, therefore require some means to make them stable. Caseins are largely confined to expression in the mammary gland, but other SCPPs are expressed in physiological fluids and hard and soft tissues. Osteopontin (OPN), in particular, is almost universally expressed in chordate tissues and biofluids (Mazzali et al. 2002). In the extracellular matrix of mammalian bone, for example, it is the major non-collagenous phosphoprotein. It is an unfolded flexible phosphoprotein, like other members of the group of SCPPs (Fedarko et al. 2004; Fisher et al. 2001; Holt et al. 2009). It contains phosphate centre-type sequences in its N-terminal half. We have previously shown that a naturally occurring mixture of OPN phosphopeptides and a purified fraction of the N-terminal phosphopeptides ending at or around residues 145 to 153 of the mature protein (Christensen and Sorensen 2014) can sequester nanoclusters of amorphous calcium phosphate (Holt et al. 2009, 2014).

### Stability diagrams for stimulated saliva, blood serum and urine

The stability diagrams for salt solutions approximating the typical compositions of three very different biofluids, urine, saliva and blood serum, have been calculated, on the assumption that OPN phosphopeptides are responsible for sequestration, and compared to experiment (Holt et al. 2014). For typical urine from non-stone formers, the critical pH is well above the physiological pH so that normally the fluid is not expected to be unstable with respect to calcium phosphate precipitation or to contain calcium phosphate nanoclusters. However, when infected by microorganisms that secrete urease, the pH may rise to above 10 and urolithiasis involving calcium phosphate stones can result.

The stability diagram of typical human saliva is difficult to calculate because the composition and pH are very variable. It is formed by mixing together different secretions from three major and many other minor glands. This biofluid is not very well buffered, so equilibration with air causes the pH to rise following the loss of CO_2_. The results for stimulated saliva, however, are more reproducible, and for this fluid, the physiological pH is above the critical pH. According to experiment and calculation, stimulated saliva contains sequestered calcium phosphate (Grøn 1973; Holt et al. 2014) although the nature and amount of the sequestering phosphoprotein or peptide are presently unknown.

The stability diagram has also been calculated for averaged blood serum (Holt et al. 2014) from healthy individuals (Walser 1961, 1962). The physiological pH is close to or a little lower than the critical pH, and because of the tight homeostasis of blood pH, we might not expect it to contain sequestered amorphous calcium phosphate. The point is particularly important for human health because vascular calcification is common in the final stages of kidney failure and is associated with fatal heart attacks. We suggested (Holt et al. 2014) that a background level of sequestering phosphopeptide was desirable to cope with the inevitable fluctuations in composition and ensure that precipitation of calcium phosphate arising from a fluctuation would be fully reversed when homeostasis is restored. The major source of phosphate centres is also uncertain with the principal candidates being OPN and fetuin A (Heiss et al. 2010; Jahnen-Dechent et al. 2008, 2011; Wald et al. 2011a, b), with contributions from other phosphoproteins (Price et al. 2003).

### Ion activity products in stimulated saliva and blood serum

Calculated saturation indices for hydroxyapatite, ACP-2 and nanoclusters sequestered by OPN peptides in fresh saliva and blood serum display a striking similarity to the calculations for milk serum (Fig. 2). The invariant p*K*_*S*_ values for fresh saliva, blood serum and a pure nanocluster solution prepared from OPN peptides are 9.26 ± 0.35, 9.27 ± 0.13 and 9.12 ± 0.05, respectively. These ion activity products are statistically indistinguishable. It is hard to escape the conclusion that milk, blood and saliva all contain similar forms of sequestered amorphous calcium phosphate, albeit at very different concentrations.

## Application to hard tissue mineralisation

The discussion so far has been concerned with the role of calcium phosphate nanoclusters in forming stable biofluids, but these structures may be de-stabilised or merely transient intermediates in the mineralisation of hard tissues. A solution of ACP nanoclusters can retain its thermodynamic stability if three conditions are satisfied (Holt et al. 2014). These are that (a) no further ACP can form, (b) the sequestered ACP does not mature into a more crystalline phase and (c) the solution is not in contact with a crystalline calcium phosphate. Condition c is violated in the mouth when saliva comes into contact with the hydroxyapatite of tooth enamel. In contact with teeth, saliva brings about remineralisation of demineralised tooth lesions. In other words, while the nanocluster solution in isolation is thermodynamically stable, when brought into contact with crystals of calcium phosphate, the crystals can grow at the expense of the nanoclusters. In effect, the solution becomes unstable and acts as a reservoir of calcium phosphate to support the growth of the more stable phase. Condition b is the most intriguing one because of the apparently conflicting literature on the roles of phosphoproteins in the inhibition and nucleation of calcium phosphate precipitation. According to the theory of calcium phosphate sequestration, the equilibrium size of the nanocluster increases as the affinity of the peptide for the surface increases. A limited amount of experimental data supports this proposition by showing that sequestration to form a stable complex requires a minimum of three phosphorylated residues in a phosphate centre and that additional, flanking, Asp residues will increase the nanocluster radius (Aoki et al. 1992; Clegg and Holt 2009). The logic is that stronger peptide binding allows bigger clusters to be sequestered, but as the cluster size increases, the probability rises of a more stable phase nucleating and growing within the cluster. This is because there are more molecules that could change in a larger cluster but also because the influence of the surface on the interior of the cluster might only be effective over a limited depth below the surface. Molecules beyond the influence of the surface can therefore behave as if they are in an unstable bulk phase. The minimum size is zero when the free energy of sequestration is zero. The largest known casein phosphate centres, and hence, possibly, the phosphate centres with the highest binding affinities, contain up to eight actual or potential phosphoseryl residues (Holt et al. 2009), but some bone and dental peptides can contain much longer phosphorylated sequences. In this context, it may be relevant that certain highly phosphorylated phosphopeptides such as phosphophoryn appear to be able to nucleate the growth of a solid phase and direct it to specific locations (Boskey et al. 2010; Gericke et al. 2010). The ability of an acidic synthetic polymer, poly-Asp, to promote intrafibrillar mineralisation of collagen has been described (Olszta et al. 2003, 2007). The mechanism involves an amorphous precursor phase which gathers near the gap zone of collagen fibres and then penetrates the fibres to form a parallel array of oriented crystals of hydroxyapatite (Nudelman et al. 2010). It has been suggested that the precursor phase is a liquid-like pre-nucleation cluster for which there is some experimental evidence in calcium carbonate nucleation studies (Gebauer and Coelfen 2011; Gebauer et al. 2008). However, we consider it more likely that, given the abundance of phosphoproteins, including osteopontin, in the extracellular matrix, the precursor phase is a form of amorphous calcium phosphate stabilised by adsorbed phosphopeptides. Indeed, it has been shown recently that the intrafibrillar mineralisation of collagen can also be accomplished by the nanocluster-forming mixture of OPN peptides (Rodriguez et al. 2014) as well as some other phosphopeptides (Nudelman et al. 2012; Sfeir et al. 2014).

## Conclusions and applications

The formation of thermodynamically stable nanoclusters of sequestered amorphous calcium phosphate is well established in cow’s milk. The casein complexes are probably responsible for most of the interspecific variation in calcium and phosphate concentrations in all milks.

The theory of calcium phosphate sequestration is developing to the point where it can make quantitative predictions of the concentrations of ions and nanocluster complexes in quite complex media including physiological fluids other than milk and proteins other than casein.

It is established that the protein OPN, which occurs in a wide distribution of species, tissues and biofluids, can also sequester amorphous calcium phosphate and form somewhat similar nanocluster complexes. The biofluids blood plasma and fresh saliva show an invariant ion activity product for nanoclusters sequestered by OPN phosphopeptides.

Amorphous calcium phosphate complexes with phosphoproteins may be formed transiently in the mineralisation of hard tissues.

There are many possible applications of the science described in this paper. These include, but are not limited to, biomimetic materials for the repair of bone fractures and demineralised teeth, artificial biofluids with superior shelf life that can be used in the irrigation of wounds during and after surgery, the irrigation of joints to help remove erosive crystals from synovial spaces, artificial serum for rapid rehydration of dehydrated patients and a superior kidney dialysis fluid that reproduces more closely the composition of blood serum. Last but not least is a growth medium in tissue engineering applications that can support the growth of mineralised tissues.
